# Induced pluripotent stem cells (iPSCs): molecular mechanisms of induction and applications

**DOI:** 10.1038/s41392-024-01809-0

**Published:** 2024-04-26

**Authors:** Jonas Cerneckis, Hongxia Cai, Yanhong Shi

**Affiliations:** 1 Department of Neurodegenerative Diseases, Beckman Research Institute of City of Hope, Duarte, CA 91010 USA; 2 Irell & Manella Graduate School of Biological Sciences, Beckman Research Institute of City of Hope, Duarte, CA 91010 USA

**Keywords:** Pluripotent stem cells, Reprogramming

## Abstract

The induced pluripotent stem cell (iPSC) technology has transformed in vitro research and holds great promise to advance regenerative medicine. iPSCs have the capacity for an almost unlimited expansion, are amenable to genetic engineering, and can be differentiated into most somatic cell types. iPSCs have been widely applied to model human development and diseases, perform drug screening, and develop cell therapies. In this review, we outline key developments in the iPSC field and highlight the immense versatility of the iPSC technology for in vitro modeling and therapeutic applications. We begin by discussing the pivotal discoveries that revealed the potential of a somatic cell nucleus for reprogramming and led to successful generation of iPSCs. We consider the molecular mechanisms and dynamics of somatic cell reprogramming as well as the numerous methods available to induce pluripotency. Subsequently, we discuss various iPSC-based cellular models, from mono-cultures of a single cell type to complex three-dimensional organoids, and how these models can be applied to elucidate the mechanisms of human development and diseases. We use examples of neurological disorders, coronavirus disease 2019 (COVID-19), and cancer to highlight the diversity of disease-specific phenotypes that can be modeled using iPSC-derived cells. We also consider how iPSC-derived cellular models can be used in high-throughput drug screening and drug toxicity studies. Finally, we discuss the process of developing autologous and allogeneic iPSC-based cell therapies and their potential to alleviate human diseases.

## Introduction

The development of induced pluripotent stem cell (iPSC) technology has opened vast opportunities for in vitro modeling of human biology and for cell therapy applications.^1–5^ Since the first reports of somatic cell reprogramming into mouse and human iPSCs in 2006 and 2007, respectively, iPSCs have been applied to model human development and diseases in vitro, screen drug candidates, and create cell therapies.^1–5^ Increasing understanding of the mechanisms that govern iPSC induction has shed light on cell fate decisions, accelerating the development of efficient iPSC derivation methods and protocols for iPSC differentiation into somatic cells.^6^ Modeling human biology with iPSCs and iPSC-derived cells is particularly attractive, given the human origin of iPSCs and the ability to derive patient-specific iPSCs with a disease-relevant genetic background.^2^ Indeed, iPSC-based cellular models may reveal human-specific phenotypes and molecular mechanisms that do not necessarily manifest in animal models.^7–9^ Furthermore, ever increasing complexity of iPSC-based cellular models has resulted in the development of sophisticated human-like tissues, such as organoids, that contain multiple cell types, exhibit primitive human tissue-like architecture and enable modeling of higher order cell-cell interactions.^10^ Various iPSC-derived cellular models can be applied to probe disease mechanisms, evaluate drug activity and toxicity, and develop next-generation cell therapies. Given that iPSCs can be genetically modified and differentiated into otherwise inaccessible cell types, autologous and allogeneic cell therapies are being actively developed using the iPSC technology and hold a great promise to provide new approaches for treating complex diseases.^11^

In this review, we begin by outlining the historical development of the iPSC technology, including the key discoveries that led to the breakthrough of somatic cell reprogramming to iPSCs in 2006 and 2007.^3–5^ Subsequently, we summarize the key molecular and cellular events governing iPSC induction as well as the methods for somatic cell reprogramming to iPSCs. We then discuss the versatile applications of iPSCs, including in vitro modeling of human development and diseases, drug discovery, and cell therapy applications.

## Historical overview of somatic cell reprogramming to iPSCs

Today, it is well established that most somatic cells harbor complete genetic information required for the development of an entire organism, whereas phenotypic diversity is achieved by epigenetic mechanisms that define gene expression potential in each cell.^12–14^ However, prior to such modern understanding of animal development, various hypotheses to explain how immense physiological complexity of an adult animal could emerge were contemplated. Popular in the 17th and 18th centuries, a theory of preformationism posited that animals would grow from miniature versions of themselves; the imagined homunculi were microscopic preformed human beings that would simply grow into their adult versions.^15^ As pioneering work in embryology accumulated and microscopy power improved, preformationism was gradually replaced by the theory of epigenesis, postulating sequential cell differentiation and organ development from an egg.^16,17^ Yet, it remained unclear how an egg cell could give rise to the breathtaking phenotypic diversity of somatic cells.

**Fig. 1:**
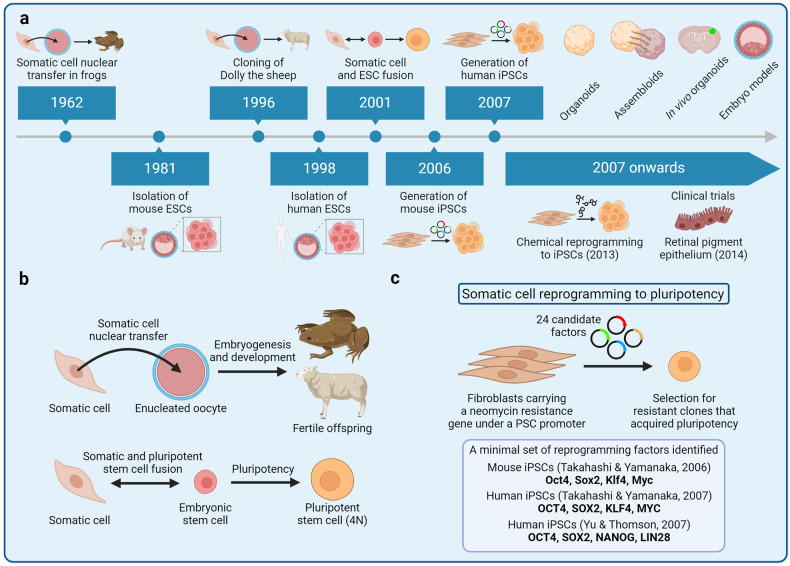
Development of the induced pluripotent stem cell (iPSC) technology. **a** A timeline of key breakthroughs related to the iPSC technology. **b** (Top) Somatic cell nuclear transfer (SCNT) experiments were pioneered by John Gurdon in the African clawed frog. Gurdon demonstrated that somatic cells retained all the genetic information necessary to give rise to a germline-competent organism. Successful SCNT in mammals was demonstrated by Keith Campbell, Ian Wilmut, and colleagues who cloned Dolly the sheep. (Bottom) Masako Tada and colleagues demonstrated that pluripotency can also be achieved by fusing a somatic cell with an embryonic stem cell, leading to the formation of a hybrid tetraploid cell. 4N, tetraploid. **c** The groundbreaking experiments of fibroblast reprogramming to pluripotency were pioneered by Kazutoshi Takahashi and Shinya Yamanaka. The researchers selected 24 factors as candidates for reprogramming and delivered these factors into mouse fibroblasts in various combinations by retroviral transduction. Eventually, Takahashi and Yamanaka identified a combination of 4 reprogramming factors—Oct4, Sox2, Klf4, and Myc—that was sufficient to reprogram mouse fibroblasts into embryonic stem cell-like pluripotent cells, known as iPSCs. Subsequently, Yamanaka and James Thomson independently reprogrammed human fibroblasts into iPSCs in 2007

In 1892, the German evolutionary biologist August Weismann (1834–1914) proposed the germ plasm theory, also known as the Weismann barrier, postulating that germ cells alone were used to transmit heritable information, whereas acquisition of somatic cell fate involved irreversible modification of heritable information, enabling phenotypic diversity to emerge.^18^ The idea of irreversible restriction of a differentiated somatic cell state during development was reiterated by the British developmental biologist Conrad Waddington (1905–1975) in 1957.^19^ Waddington proposed a model that would become known as the Waddington’s epigenetic landscape, suggesting that cell differentiation resembled a ball rolling downhill towards a more and more restricted and irreversible state.^19^ However, it remained elusive whether somatic cell differentiation truly required irreversible mutational events to occur or whether it could be achieved by some other means, such as by reversible epigenetic mechanisms.^14^ A year later, the American geneticist David Nanney (1925–2016) proposed that while the DNA sequence conferred gene expression potential, phenotypic differences in cells sharing the same genome could arise because of gene expression “specificities” regulated by epigenetic systems.^20^ Indeed, the reversibility of the mechanisms governing somatic cell specification was demonstrated by the British developmental biologist John Gurdon (b. 1933), who performed somatic cell nuclear transfer (SCNT) experiments (Fig. 1a, b).^21–25^ In 1962, using a model of the *Xenopus laevis* frog, Gurdon demonstrated that a nucleus isolated from a terminally differentiated somatic cell and transplanted into an enucleated egg harbored all the genetic information required to give rise to germline-competent organisms.^21–24^ Therefore, the SCNT experiments revealed that genetic information was preserved during differentiation, whereas phenotypic diversity of somatic cells was likely achieved by reversible epigenetic mechanisms. What kind of epigenetic mechanisms could enable such elaborate yet reversible phenotypic diversity? Among the many layers of epigenetic regulation known today, DNA methylation is a prominent example of stable, yet reversible epigenetic memory acquired along the course of cell fate specification.^26–29^ For a historical review of discovering DNA methylation as a central mechanism of gene expression regulation and maintenance over mitotic divisions, the readers are referred to Tompkins, 2022.^14^

In 1981, British biologists Martin Evans (b. 1941) and Matthew Kaufman (1942–2013) as well as the American biologist Gail Martin (b. 1944) isolated mouse embryonic stem cells (ESCs) that would serve as a reference point for subsequent somatic cell reprogramming experiments.^30,31^ Human ESCs were isolated by the American developmental biologist James Thomson (b. 1958) and colleagues in 1998.^32^ Cell fusion experiments of mouse^33^ and human^34^ ESCs with somatic cells revealed the capacity of the resulting heterokaryon for reprogramming to pluripotency, thus reaffirming the notion of cellular plasticity and somatic cell fate reversibility observed by Gurdon (Fig. 1b). Transdifferentiation experiments by ectopic expression of transcriptions factors further revealed the importance of transcription factors in establishing cell fate; for example, overexpression of the C/EBPα/β transcription factors was found to promote B cell reprogramming into macrophages.^35–38^ With ESCs as a reference point for features of pluripotency and an emerging understanding of how transcription factors orchestrated gene expression, the Japanese stem cell biologist Shinya Yamanaka (b. 1962) together with his postdoctoral fellow Kazutoshi Takahashi designed a series of somatic cell reprogramming experiments that would lead to the breakthrough development of mouse iPSCs in 2006 (Fig. 1c).^4^ Aiming to induce pluripotency in mouse embryonic fibroblasts (MEFs), Takahashi and Yamanaka selected 24 potential reprogramming factors that included transcription factors known to be important for the ESC state and other effectors. The reprogramming factors were cloned into retroviral vectors for MEF transduction, whereas MEFs were engineered to carry β-galactosidase and neomycin resistance encoding genes under a pluripotency-specific promoter of the *Fbxo15* gene. Screening different combination of the 24 reprogramming factors, Takahashi and Yamanaka narrowed down the list to four transcription factors that were sufficient to induce pluripotency in MEFs: Oct4, Sox2, Klf4, and Myc (together known as OSKM or Yamanaka factors).^4^ Remarkably, these mouse iPSCs resembled the biological potency, gene expression, and the epigenetic landscape of ESCs.^39^ A year later, Yamanaka and Thomson independently demonstrated that human fibroblasts could also be reprogrammed into iPSCs; Yamanaka used the same OSKM factors, whereas Thomson used OCT4, SOX2, NANOG, and LIN28.^3,5^ These combinations of reprogramming factors remain widely used today, whereas Gurdon and Yamanaka were awarded the 2012 Nobel Prize in Physiology or Medicine for their discoveries. Since 2007, various modifications to the original cocktail of reprogramming factors have been developed. For example, small-molecule assisted somatic cell reprogramming was first reported in 2008,^40,41^ whereas fully chemical reprogramming of murine fibroblasts using seven small-molecule compounds was achieved in 2013.^42^

## Molecular mechanisms of somatic cell reprogramming to iPSCs

When pluripotent stem cells undergo differentiation into somatic cells, they acquire epigenetic memory and undergo global changes to their chromatin conformation, resulting in inactivation of pluripotency-specific genes and activation of somatic cell-specific genes.^43^ Reprogramming of somatic cells back to the pluripotency state involves the erasure of many of these somatic cell signatures; therefore, induction of pluripotency has been proposed to partially resemble the a sequence of developmental events in reverse.^6,44–46^ Broadly, reprogramming occurs in two phases, early and late. During the early phase, somatic genes are silenced, whereas early pluripotency-associated genes are activated; during the late phase, late pluripotency-associated genes are activated. Early events of reprogramming are largely stochastic, presumably owing to the inefficient access of closed chromatin by OSKM and other transcription factors, whereas late events appear to be more deterministic.^6^ Universal aspects of reprogramming, such as two transcriptional waves, are accompanied by somatic cell type-specific reprogramming trajectories and transient events.^47^ Overall, reprogramming entails profound remodeling of the chromatin structure and the epigenome as well as changes to almost every aspect of cell biology, including metabolism, cell signaling, intracellular transport, proteostasis, and others.^48–52^ Given that iPSCs are most often derived from fibroblasts, mesenchymal-to-epithelial transition (MET) is another critical event that occurs during reprogramming.^53^

Uncovering the molecular mechanisms of iPSC induction facilitates the development of novel reprogramming approaches and reveals the underlying principles of cell fate transitions and cell fate determination. This knowledge can subsequently be used to design rational strategies for iPSC differentiation towards the desired cell types in an efficient manner. In this section, we focus on the roles of transcription factors as well as chromatin and DNA methylation dynamics in reprogramming.

### Transcription factors

OSKM and other transcription factors orchestrate somatic cell reprogramming to pluripotency.^54,55^ Through concerted action, OSKM expel somatic cell-specific transcription factors from somatic enhancers and activate pluripotency enhancers; silencing of somatic cell-specific enhancers is initiated early in reprogramming, whereas activation of pluripotency-specific enhancers occurs later in reprogramming.^56,57^ Notably, the chromatin and DNA methylation landscape is restrictive early in reprogramming, requiring pioneering activity of the OSKM factors to access closed chromatin and initiate gene expression.^58^ Oct4, Klf4, and Sox2 target partial motifs in the nucleosome-enriched loci, indicating their pioneering activity,^55^ whereas Sox2 has even been proposed to be a super pioneer due to its ability to induce DNA demethylation and overcome repressive epigenome.^59^ Multiple studies have revealed the dynamics of OSKM binding to DNA and their mode of action. For example, Oct4 dynamics exhibit a hierarchical sequence of events, with Oct4 targeting epigenetically primed states and then maintaining stable DNA occupancy for the duration of reprogramming.^56^ Mutagenesis-based analysis of Oct4 protein domains has revealed dynamic DNA and nucleosome binding kinetics and highlights the importance of stable Oct4 interactions with nucleosomes to maintain chromatin accessibility of pluripotency enhancers.^60^ Klf4 facilitates topological enhancer-promoter connectivity and organization required for reprogramming to pluripotency,^61^ whereas Myc targets open promoter regions to facilitate cell cycle progression.^6,62,63^ Importantly, OSKM closely cooperate with each other to exert global reprogramming of gene expression, which can be illustrated by the concerted action of OSKM to drive MET: Oct4 and Sox2 suppress *Snail* expression, Klf4 promotes *Chd1* expression (encoding E-cadherin), and Myc suppresses the TGFβ signaling axis.^64^ In addition to OSKM, multiple other transcription factors play important roles in reprogramming downstream of OSKM and can partially substitute certain OSKM factors.^65–69^ For example, Klf4 and Sox2 can be substituted by their close homologs,^6,70^ whereas NKX3-1 or a dominant-negative variant of c-Jun can substitute Oct4.^67,71^ Notably, certain cell types that endogenously express SKM, such as neural progenitor cells, can be reprogrammed into iPSCs with exogenous expression of Oct4 alone.^72–74^ Overall, transcription factors are the drivers of somatic cell reprogramming to pluripotency that coordinate the rewiring of gene expression as well as the remodeling of chromatin and DNA methylation as discussed next.

### Chromatin dynamics and histone remodeling

Chromatin remodeling represents another layer of dynamic changes that occur during reprogramming.^44,75,76^ Although pioneer transcription factors can access closed chromatin, the ability of non-pioneer transcription factors to exert gene expression programs requires extensive chromatin remodeling. Given that chromatin becomes progressively restricted during cell differentiation to establish somatic cell-specific gene expression programs,^43^ decompaction and remodeling of chromatin is essential for induction of pluripotency. Chromatin remodeling often precedes changes in gene expression and is required for establishing pluripotency-supporting spatial organization of DNA regulatory elements as well as for enabling access of transcription factors to DNA during reprogramming.^45,77^ Chromatin remodeling occurs in waves as loci enriched for somatic genes transition from open to closed early in reprogramming, whereas loci enriched for OSK motifs transition from closed to open late in reprogramming.^75,78^

Chromatin dynamics are highly influenced by nucleosome remodeling and histone modifications that modulate chromatin compaction and transcription factor accessibility to DNA. Nucleosome remodeling factors, such as the NuRD complex and the histone chaperone CAF-1, exert context-dependent regulation of gene expression in somatic cells and during induction of pluripotency.^79,80^ For example, CAF-1 is required for maintaining somatic cell identity, whereas suppression of *CAF-1* facilitates chromatin opening at enhancer regions and promotes Sox2-mediated activation of pluripotency genes.^79^ Various histone modifiers are also involved in reprogramming; for example, the histone methyltransferase EZH2 is a positive regulator of reprogramming, presumably required to silence somatic cell-specific genes.^81^ On the other hand, histone methyltransferase DOT1L is a negative regulator of reprogramming because it maintains permissive chromatin in fibroblast-specific genes associated with the epithelial-to-mesenchymal transition.^81^ Changes in global levels of specific histone modifications have also been documented in reprogramming. For example, H3K9 methylation is depleted in iPSCs, and suppression of the H3K9 reader heterochromatin protein *Cbx3* promotes fibroblast reprogramming to pluripotency.^82,83^ Global remodeling of histone modifications can be driven by metabolic reprogramming during the induction of pluripotency. For example, the transcription factor Glis1 targets glycolytic genes to enhance glycolytic flux during reprogramming, leading to increased production of acetyl-CoA and lactate intermediates required for histone acetylation and lactylation at pluripotency genes.^84^ Given the roles of histone modifiers in chromatin compaction and reprogramming, small-molecule compounds targeting histone modifiers are often used to promote chromatin decompaction during chemical or transcription factor-mediated reprogramming. For example, the histone deacetylase inhibitor valproic acid as well as the Dot1l inhibitor SGC0946 promote somatic cell reprogramming to pluripotency.^40,85,86^

### DNA methylation

Given the critical role of DNA methylation in establishing epigenetic memory during cell differentiation, active remodeling of DNA methylation is another essential part of reprogramming. In development, DNA cytosine methylation is orchestrated by de novo DNA methyltransferases DNMT3A/B that guide DNA methylation at regulatory regions, thus modulating transcription factor accessibility and downstream gene expression.^87,88^ During reprogramming, such somatic cell-specific DNA methylation patterns are reversed by active DNA demethylation mediated by ten-eleven translocation (Tet) enzymes.^89–91^ Indeed, waves of global DNA demethylation during reprogramming result in the loss of DNA methylation at regulatory regions that become enriched for 5-hydroxymethylcytosine (5hmC), an intermediate of Tet-mediated DNA demethylation.^92–95^ These actions of Tet enzymatic activity not only facilitate pluripotency-specific gene expression, but also drive other events required for reprogramming, including MET.^96^ Furthermore, Tet enzymes target specific loci to facilitate reprogramming; for example, Tet1 demethylates the endogenous Oct4 locus to reactivate Oct4 expression.^97,98^ Tet1 can even substitute exogenous Oct4 during reprogramming, indicating a central role for active DNA demethylation in reprogramming to pluripotency.^98^ Tet enzymes cooperate with pluripotency-specific transcription factors to reactivate pluripotency-specific genes. For example, Nanog physically interacts with Tet1 and Tet2, whereas cooperative binding of Nanog and Tet1 to loci of pluripotency-specific genes primes their expression during reprogramming.^97^ Tet1 activity is also influenced by exogenous vitamin C, indicating that small-molecule compounds can influence active DNA demethylation and epigenetic remodeling during reprogramming.^99^ Overall, remodeling of chromatin accessibility and DNA methylation erases somatic cell identity and creates a permissive epigenetic landscape for the pluripotency state during reprogramming.

### Population-level dynamics during iPSC induction

The dynamics of cell fate transitions at the population level reveal a stochastic and heterogenous nature of iPSC induction.^76^ Somatic cells transition through a continuum of reprogramming intermediates that bifurcate into intermediates that will successfully complete reprogramming and those that will acquire an alternative fate.^100^ Most cells do not complete reprogramming, whereas clonal competition leads to the emergence of dominant clones that overtake the culture during reprogramming.^101^ Clonal competition is also fueled by the heterogeneity of the starting somatic cell population, the extent of which may be dependent on the somatic cell source.^101^ There is a great interest in isolating rare intermediates that complete reprogramming more efficiently than do other cells, so that molecular mechanisms governing productive reprogramming could be elucidated.^102^ For example, rare intermediates that exhibit chromatin hyperaccessibility at pluripotency-specific genes and distinct DNA methylation profiles have been isolated based on the presence of pluripotency-specific surface markers.^103^ We anticipate that improving high-throughput profiling of gene expression and chromatin accessibility at single cell level will continue to provide new insights into cell fate transitions and reprogramming trajectories during iPSC induction.

### Residual somatic cell memory and reprogramming cell source

Although iPSCs resemble primary ESCs in terms of their cellular characteristics and the potential for differentiation into all lineages, limitations associated with reprogramming and persistent features of somatic cell identity render iPSCs distinct. Reprogramming of various somatic cell types reveals persistence of somatic cell transcriptional, DNA methylation, and chromatin accessibility signatures.^104–107^ Incomplete removal of somatic cell-specific epigenetic signatures as well as aberrant de novo DNA methylation associated with reprogramming can affect the status and the differentiation potential of iPSCs.^105,107,108^ Adding small-molecule compounds that target chromatin modifiers to the reprogramming cocktail can facilitate the erasure of the residual chromatin signatures and increase the differentiation potential of iPSCs into alternative lineages.^108^ On the other hand, persistence of somatic cell-specific epigenetic signatures can be exploited to enhance iPSC differentiation into the desired cell type by deriving iPSCs from the same somatic cell type. For example, iPSCs derived from pancreatic beta cells retain open chromatin signatures at loci important for beta cell identity; consequently, beta cells can be differentiated more efficiently from beta cell-derived iPSCs as compared to non-beta-cell-derived iPSCs.^104^

The cell source used for reprogramming can also influence the heterogeneity and the mutational burden of the resulting iPSCs. iPSCs derived from skin fibroblasts contain common ultraviolet (UV) light-related mutations and exhibit genomic heterogeneity, likely arising from the already heterogenous fibroblast population of the skin.^109^ On the contrary, iPSCs derived from peripheral blood mononuclear cells (PBMCs) do not exhibit UV-related damage and may have fewer mutations than do iPSCs derived from skin fibroblasts. Nonetheless, PBMC-derived iPSCs may contain other mutations that are selected for during reprogramming, such as oncogenic mutations in the *BCOR* gene encoding the BCL-6 corepressor.^109^ Age-related heteroplasmic variants of mitochondria can also influence the mitochondrial genetic makeup of iPSCs derived from different donors.^110^ Furthermore, spontaneous mutations that arise in the mitochondrial genome during reprogramming could result in the production of novel immunogenic epitopes; new iPSC-specific mitochondrial DNA mutations have been observed in >70% of iPSC lines.^110,111^ Overall, iPSCs exhibit increased heterogeneity as compared to ESCs due to persistent somatic cell signatures and mutational burden.^112^ Such heterogeneity can influence the quality of iPSCs, including their differentiation potential and the immunogenicity of iPSC-derived cellular products, among other features.

## Methods of iPSC induction

Since the groundbreaking experiments of fibroblast reprogramming into iPSCs, various approaches to deliver reprogramming factors into somatic cells and induce pluripotency have been developed.^113–115^ Viral vectors carrying OSKM expression cassettes are commonly used for reprogramming due to their high efficiency of infection and the capacity to transduce various somatic cell types.^3–5,113,115–119^ Viral vectors can be classified as either integrating or non-integrating vectors; lentiviral or retroviral delivery of the reprogramming factors leads to their integration into the genome and thus stable expression for iPSC induction.^3–5^ However, viral vector integration into the genome may result in insertional mutagenesis and undesired transgene reactivation beyond the duration of reprogramming. An alternative approach is to use non-integrating viral vectors, such as adenovirus, adeno-associated virus, or Sendai virus.^115,119^ Non-integrating viral vectors are gradually cleared from proliferating iPSCs, resulting in reprogramming without permanent OSKM integration or disruption of the genome. OSKM factors can also be delivered using non-viral vectors, such as transposons,^120,121^ episomal plasmids,^122,123^ mRNA,^124^ and others.^115^ For example, plasmid-based episomal vectors are commonly used to derive iPSCs for clinical development; reprogramming efficiency when using episomal vectors is comparable to that of Sendai virus-mediated reprogramming, but the cost is much lower.^122,123,125^ Somatic cells can also be reprogrammed into iPSCs without OSKM overexpression. Various combinations of miRNAs can be used to activate the endogenous pluripotency gene networks.^126,127^ For example, human and mouse iPSCs can be derived by overexpression of *miR-200c*, *miR-302s*, and *miR-369s*.^127^ Alternatively, pluripotency can be induced using a cocktail of small-molecule compounds that modulate various signaling pathways and epigenetic modifiers.^128^ Small-molecule-based chemical reprogramming is highly attractive due to its simplicity and potential for scalability.^128–130^ Combining transcription factors and small-molecule compounds may further accelerate reprogramming.^131–133^ Overall, the desired method is often selected based on its efficiency, feasibility, safety, and cost.^115^

It should be noted that new insights into the molecular mechanisms of reprogramming using the methods described above are constantly emerging. For example, chemical reprogramming is associated with distinct cell fate transitions and chromatin accessibility dynamics as compared to transcription factor-mediated reprogramming, but it remains unclear if such differences affect the status of the derived iPSCs.^134,135^ Furthermore, aberrant Oct4 off-target activity has been linked to changes in gene expression and epigenetic profiles that may alter the iPSC differentiation potential.^136^ Therefore, newly developed reprogramming methods should be rigorously assessed for their effects on the iPSC status, quality, and differentiation potential.

## Applications of iPSCs

Development of the iPSC technology has transformed in vitro research and therapeutic development.^2,137^ iPSCs can proliferate almost indefinitely and be differentiated into the diversity of human cell types, but with reduced ethical constraints as compared to using human ESCs.^138,139^ As a result, iPSC-derived cells are widely used for modeling human development and diseases, performing high-throughput drug screening, and developing autologous and allogeneic cell therapies, among other applications. In the rest of the review, we discuss the diverse applications of iPSCs, their key advantages, as well as the limitations that remain to be overcome.

## iPSC-derived cellular models

Assembling cellular models of human development and diseases in vitro requires access to large quantities of cells that faithfully recapitulate human biology. Although various primary cell types, such as skin, blood, and cancer cells, can be easily isolated from living donors, other cell types, such as brain and heart cells, are largely unavailable. An alternative approach is to use rodent cells; however, animal models exhibit substantial species divergence and may not recapitulate certain human-specific phenotypes.^7–9^ The iPSC technology can be used to overcome both limitations: iPSCs can be readily differentiated into hard-to-access cell types, whereas their human origin and relevant genetic background enable robust modeling of human biology in vitro.

**Fig. 2:**
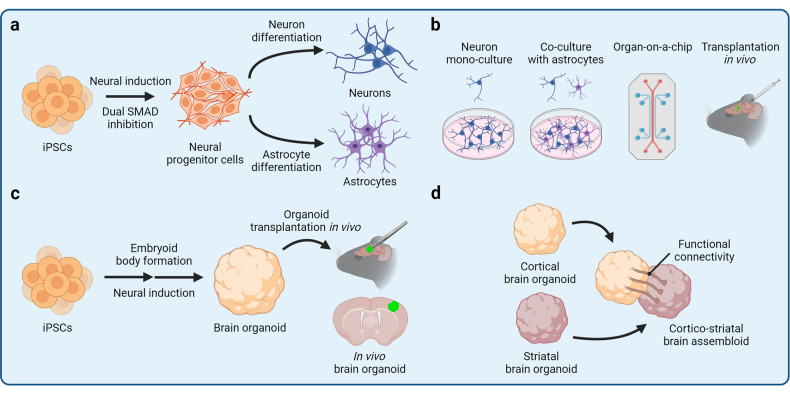
Induced pluripotent stem cell (iPSC)-derived cellular models. The iPSC technology can be applied to derive cellular models of varying complexity, ranging from two-dimensional mono-cultures to three-dimensional multicellular assemblies. Various neural cellular models are shown as an example. **a** Differentiation of neural progenitor cells (NPCs) from iPSCs is achieved by promoting neuroectoderm specification by dual SMAD inhibition. Subsequently, NPCs can be differentiated into terminal neural lineage cells, such as neurons and astrocytes. **b** iPSC-derived cells can be maintained in a mono-culture or together with other cell types in a co-culture. Different cell types can also be assembled into an organ-on-a-chip that contains separate compartments and enables modeling of complex tissue architecture. Alternatively, iPSC-derived cells can be transplanted in vivo to expose the cells to a complex tissue environment. **c** iPSCs can be differentiated into three-dimensional self-organizing organoids that partially resemble endogenous tissue architecture and contain several cells types. Organoids can also be transplanted in vivo to promote their vascularization and maturation. **d** Different types of organoids can be fused together into assembloids for the study of higher-order tissue interactions, such as long-distance innervation and cell migration

To date, hundreds of protocols to differentiate iPSCs into various cell types have been developed. This is often achieved by mimicking developmental signaling cues in vitro with relevant proteins and small-molecule compounds or by overexpression of cell fate-determining transcription factors to instruct the desired gene expression programs. Certain cell types, such as neurons or cardiomyocytes, can be differentiated with limited resources and training required in about one week.^140,141^ Other cell types, such as oligodendrocytes or T cells are more difficult to differentiate and require extensive technical expertise.^142–144^ For example, differentiation of oligodendrocytes, which arise late in human brain development, involves multiple stages, requires several different media formulations, and can take several months.^143–145^ Approaches for uncovering key effectors required for efficient cell differentiation include CRISPR/Cas9-based screens, temporal high-throughput profiling of differentiation trajectories, and comprehensive annotation of transcription factor activity, among others.^146–149^ In-depth understanding of developmental trajectories facilitates rational design of differentiation protocols to derive specific cell types and subtypes. For example, hematopoietic lineage cells can be derived by sequential specification of the mesoderm and the hemogenic endothelium to obtain hematopoietic progenitor cells followed by terminal differentiation of lymphoid and myeloid lineages in the presence of relevant cytokines.^150,151^ Neural cells can be derived by dual SMAD inhibition that promotes neuroectoderm specification and the emergence of neural progenitor cells (Fig. 2a).^152,153^ Furthermore, various morphogens can be applied to instruct regional identity of the differentiating neural cells to obtain specialized cell subtypes; for example, inhibition of the WNT signaling pathways specifies forebrain identity of neural cells.^153^ iPSC differentiation can also be considerably accelerated by ectopic expression of cell fate-determining transcription factors. For example, overexpression of six microglia fate-determining transcription factors facilitates rapid differentiation of iPSCs into microglia in as few as 8 days, as compared to several weeks required for microglia differentiation without the use of transcription factors.^154^

Cellular models of varying complexity can be assembled from iPSC-derived cells (Fig. 2b). A particular cell type can be studied in mono-culture experiments to evaluate the cellular response to experimental perturbations and uncover cell autonomous molecular mechanisms and phenotypes. Due to its simplicity, mono-culture is also often used to perform high-throughput screens, such as CRISPR/Cas9-based screens, high-content imaging, and drug screening.^155–157^ However, the mono-culture environment lacks heterotypic paracrine signaling and cell-cell interactions that are indispensable in vivo. To increase the complexity of iPSC-derived in vitro models, different cell types can be co-cultured together. Co-culture not only enables the study of cell-cell communication, but also promotes cell maturation. For example, co-culturing neurons with astrocytes enhances neuron maturation and survival because astrocytes provide neurotrophic factors required for neuron maintenance.^140^ Tri-culture of neurons, astrocytes, and microglia further increases the physiological relevance of the in vitro brain model, enabling complex phenotypes to emerge.^158,159^ Yet, co-culture experiments still lack the three-dimensional (3D) complexity and organization of human tissues. Remarkably, iPSCs have the capacity to self-organize into 3D tissues, known as organoids, if appropriate differentiation conditions are provided (Fig. 2c).^10,160–164^ Organoids are often comprised of several cell types and partially recapitulate the complexity of human tissues, enabling the study of context-dependent cell function, organogenesis, and organ-specific diseases. The organoid field has grown extensively in recent years, and dozens of protocols have been developed to derive organoids representing major human organs.^10,160–164^ Importantly, organoids can develop impressive complexity; brain organoids patterned by Sonic hedgehog (SHH) signaling exhibit human-like topographical specification with neocortical, ganglionic eminence, and hypothalamic regions.^165^ Kidney organoids contain nephron-like segments, including the Bowman’s capsule, proximal tubules, the loop of Henle, and distal convoluted tubules in a continuous arrangement reflective of the human kidney architecture.^166^ Increasing sophistication of organoid differentiation protocols also enables derivation of organoids resembling specific organ regions. For example, exposure of developing neural organoids to various combinations of patterning morphogens yields cortical,^167,168^ midbrain,^169,170^ hippocampal,^171^ cerebellar,^172,173^ retinal,^174–176^ and other specialized brain organoids.^177–180^ Similarly, fundic and antral gastric organoids recapitulate distinct epithelial lining of the corpus and antrum regions of the stomach, respectively.^181,182^ Organoid complexity can be further increased by developing multi-lineage organoids or fusing heterotypic organoids to form assembloids (Fig. 2d).^183–185^ For example, multi-lineage neuromuscular organoids contain both neurons and skeletal muscle cells and thus form functional neuromuscular junctions.^186^ Similarly, fusing cortical organoids with spinal cord organoids and skeletal muscle spheroids results in the formation of corticofugal projections and innervation of the muscle tissue.^187^

An alternative platform to self-organizing organoids is the organ-on-a-chip (OoC), a biomimetic assembly of tissue-relevant cell types into a microfluidics device to recapitulate certain aspects of tissue architecture.^188–194^ OoCs have separate compartments and are constantly perfused, enabling controlled tissue assembly, exposure to shear fluid forces, and separation of culture medium reservoirs. OoCs can be used to model tissue interfaces, such as the blood-brain barrier (BBB)^195,196^ or the airway epithelium,^197^ where compartment separation is critical. Assembling iPSC-derived neural cells and brain microvascular endothelial-like cells (BMECs) into a BBB-on-a-chip yields a BBB model that exhibits in vivo-like transendothelial electrical resistance and restricted permeability.^198^ As a result, the BBB-on-a-chip can be perfused with whole human blood at the BMEC interface without inducing toxicity in the neural cell compartment.^198^ Microfluidics devices can also be designed to incorporate other functional elements, such as valves to support the mechanical function of cardiac tissue. Fabrication of a microfluidics system with valves has been used to establish an iPSC-derived heart-on-a-chip with unidirectional fluid flow and a closed pressure-volume loop.^199^ Heart-on-a-chip devices can record various parameters of cardiac function, including contractile dynamics, active force, tension, and electrical properties of the engineered tissue.^200^

iPSC-derived cells and organoids can also be transplanted in vivo to obtain humanized animal models (Fig. 2c).^201–205^ In this way, the advantages of iPSC-derived cells, including their human origin and donor-specific genetic background, can be combined with the advantages of animal models, such as their physiological complexity, ability to exhibit cognitive phenotypes, and others. For example, transplantation of iPSC-derived microglia into the mouse brain leads to even distribution of microglia in the brain parenchyma, improved maturation, and long-term survival of microglia.^206–210^ Similarly, blood vessel organoids form perfusable vascular networks upon transplantation, which is challenging to achieve in vitro.^211^ Overall, iPSC-derived cellular models of varying complexity can be generated to address specific hypotheses of cellular function, cell-cell interactions, and tissue-level activity.

## Maturation of iPSC-derived cells

Differentiation of iPSCs into various cellular models, especially in mono-culture, occurs with limited exposure of the differentiating cells to a physiologically-relevant tissue microenvironment and at an accelerated rate as compared to cell differentiation in vivo. As a result, iPSC-derived cells are often immature, which is a significant limitation of the iPSC technology to disease modeling and cell therapy applications. Immature cells lack complete functionality of their in vivo counterparts and thus may not reveal important phenotypes when used for disease modeling or be as efficacious as primary cells when used in cell therapy. For example, immature iPSC-derived spinal motor neurons exhibit fetal-like signatures, whereas expression of gene networks relevant to amyotrophic lateral sclerosis (ALS) correlates with motor neuron maturation and aging; these observations suggest that immature iPSC-derived neurons may not fully recapitulate ALS pathology.^212^ Therefore, achieving robust maturation of iPSC-derived cells is an important consideration before downstream applications are pursued.

Somatic cells differentiate and mature in the context of their tissue microenvironment that provides signaling cues, metabolites, and cell-cell contacts required for maturation. Reconstituting a physiologically-relevant environment in vitro can thus promote maturation of iPSC-derived cells. For example, artificial extracellular matrix composed of biomimetic nanofibers enhances cortical neuron morphological and functional maturation.^213^ Relevant paracrine signaling can also be provided by co-culture experiments, where two or more cell types interact with each other. Co-culture of cardiomyocytes with mesenchymal stem cells promotes myofibril alignment and gap junction formation in cardiomyocytes.^214^ Such enhanced cardiomyocyte maturation is partially mediated by mesenchymal stem cell secreted extracellular vesicles, highlighting the importance of paracrine cell-cell interactions that would be challenging to replicate using chemically defined cell culture medium alone.^214^ That cell-cell interactions promote maturation of iPSC-derived cells is also evident in 3D in vitro cellular assemblies, including organoids and OoCs that generally exhibit improved maturation over 2D cellular models. For example, incorporating cardiac fibroblasts into spheroids containing cardiomyocytes and epithelial cells leads to cardiomyocyte-fibroblast coupling via gap junctions as well as enhances sarcomere formation and cardiomyocyte eletrophysiological maturation.^215^ Similarly, a BBB-on-a-chip exhibits metabolic coupling between neurons and endothelial cells.^216^ Organoid maturation can be further improved by transplantation in vivo, leading to organoid vascularization, improved nutrient exchange, and exposure to physiologically-relevant systemic factors.^217–223^ For example, orthotopically transplanted lacrimal gland organoids functionally mature to produce tear-film proteins and resemble primary human tissue.^217^

Somatic cells are also exposed to tissue-specific mechanical and environmental conditioning, which may be partially recreated in vitro. Application of mechanical stress to iPSC-derived cardiomyocytes by stretching improves their transcriptional and functional maturation.^224,225^ Incremental pulsatile stretching also promotes maturation of vascular grafts composed of iPSC-derived smooth muscle cells, leading to increased mechanical strength and minimized dilation of the engineered vessels.^226^ Fluid shear stress enhances ciliogenesis and maturation of multiciliated airway cells, whereas cardiomyocyte maturation can be further improved by electrical field conditioning.^197,200,227^ Overall, paracrine signaling and mechanical cues can be readily applied to achieve advanced maturation of iPSC-derived cells.

Ultimately, iPSC-derived cells should faithfully recapitulate the cellular biology and function of their in vivo counterparts to serve as rigorous in vitro models of human development and diseases. Large omics datasets generated from primary human tissues can be used for benchmarking of iPSC-derived cells to determine their maturity and resemblance to primary cells. For example, Shin et al. performed spatial similarity mapping of single-cell transcriptomes of iPSC-derived thalamic organoids and primary human brain tissue, which revealed a strong resemblance of thalamic organoids to the primary thalamus.^228^ Therefore, efforts to generate multi-omics datasets of primary tissues, such as the Human Cell Atlas Project,^229,230^ can provide highly valuable data for iPSC-based studies and serve as a reference point for molecular profiles of functionally mature cells and tissues.

## Modeling human development with iPSC-derived cells

Given that iPSCs resemble an ESC-like state after reprogramming,^39^ iPSC differentiation into somatic cells or organoids primarily recapitulates embryonic developmental and fetal-like cell states. Therefore, iPSCs are particularly suitable for modeling early human development. Controlled differentiation of iPSCs recapitulates key events of early embryogenesis, such as epiblast lumenogenesis, bipolar embryonic sac formation, and specification of the primitive streak and primordial germ cells.^231–234^ iPSC-derived primordial germ cell-like cells (PGCLCs) exhibit distinct germline-specific transcriptional programs and can be used to study germline development.^232,234^ Furthermore, differentiation of iPSCs towards presomitic mesoderm recapitulates human somitogenesis and the phenomenon of the segmentation clock.^235^ Recently, derivation of post-implantation human embryo models from ESCs has been reported.^236^ We anticipate that iPSCs will soon be applied to derive such sophisticated embryo models as well.^237^

Although human iPSCs resemble the post-implantation epiblast, they can also be reprogrammed into naïve iPSCs that resemble the pre-implantation epiblast to study human embryogenesis before blastocyst implantation.^238–240^ Derivation of naïve human iPSCs from somatic cells was first reported in 2009 and generally requires a combination of transcription factors and small-molecule compounds that modulate various signaling pathways.^240–242^ Naïve iPSCs can be used to study X chromosome inactivation, dynamics of transposable element regulation, cell fate transitions, extraembryonic lineage differentiation, and other features and events of pre-implantation embryogenesis.^240,243,244^ Blastoid organoids have been recently developed from naïve iPSCs to study blastocyst development and implantation.^245^ In addition to naïve iPSCs, trophoblast stem cells can be derived from iPSCs to model placental development.^246–248^

Differentiation of iPSCs into specific cell types reveals the principles of cell type specification and maturation. For example, profiling of dopaminergic neuron differentiation trajectories by single-cell RNA sequencing (scRNA-seq) has indicated an important role for the ASCL1 transcription factor in dopaminergic neuron specification.^249^ Differentiation of multiple iPSC lines can also be used to conduct population level analyses, such as the quantitative trait loci (QTL) analysis.^250^ In this way, gene regulatory mechanisms that play important roles in development may be uncovered. The organoid platform can be used to study the development of distinct organs. For example, temporal high-throughput profiling of brain organoid differentiation reveals transcriptional and epigenetic regulomes that orchestrate human brain development and regionalization of different brain areas.^251,252^ Spinal cord organoids recapitulate certain features of neural tube development by undergoing neurulation-like morphogenesis,^253^ whereas cardiac organoids co-cultured with epicardial-like cells mimic the envelopment of the myocardium by the epicardium that occurs during heart development.^254^ Finally, assembloids enable modeling of multi-tissue interactions that shape developmental programs through paracrine signaling and cell migration.^255^ For example, fusing anterior and posterior gut spheroids leads to the emergence of a hepato-biliary-pancreatic anlage-like structure at the interface of the two spheroids in a process that is regulated by retinoic acid signaling.^256^ Heterotypic brain assembloids, such as cortico-striatal assembloids, recapitulate interneuron migration that occurs during brain development as well as formation of long-range neuronal projections (Fig. 2d).^187,257,258^ Overall, modeling development with iPSC-derived cells can provide important insights into human-specific developmental programs and inform cell differentiation approaches for other applications as discussed next.

## Modeling human diseases with iPSC-derived cells

**Fig. 3:**
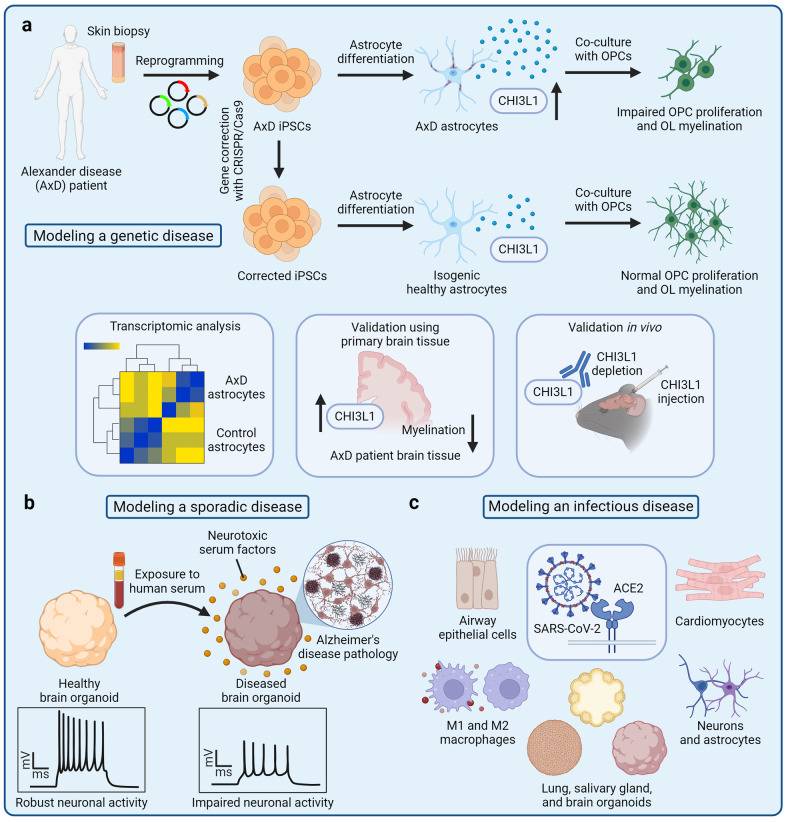
Disease modeling with iPSC-derived cells. **a** Genetic diseases, such as Alexander disease (AxD), can be modeled using patient-derived iPSCs that carry disease-causing mutations.^144^ A tissue biopsy is first taken from a patient with AxD. Somatic cells are reprogrammed into iPSCs, and the *GFAP* mutations that cause AxD are corrected by gene editing. Patient-derived iPSCs and isogenic corrected controls are then differentiated into astrocytes that express *GFAP* at high levels. Co-culture of AxD astrocytes with oligodendrocyte progenitor cells (OPCs) reveals impaired OPC proliferation and oligodendrocyte (OL) myelination. Transcriptomic analysis indicates increased expression of the *CHI3L1* gene, whereas OPC dysfunction can be partially reversed by CHI3L1 protein depletion. These observations in vitro can be further validated in primary human brain tissues as well as and experiments in vivo. **b** Sporadic diseases, such as Alzheimer’s disease (AD), can be modeled with patient-derived iPSCs that harbor genetic risk factors; alternatively, iPSC-derived cells can be exposed to non-genetic risk factors to induce disease-relevant pathology. For example, exposure of iPSC-derived brain organoids to human serum mimics the breakdown of the blood-brain barrier and induces AD-like pathology. Brain organoids exposed to neurotoxic serum factors have increased levels of toxic amyloid peptides and hyperphosphorylated tau as well as exhibit impaired neuronal activity. **c** Infectious diseases, such as COVID-19, can be modeled by exposing iPSC-derived cells and organoids to viral pathogens. iPSC-based models of viral infection can reveal human-specific tropism, mechanisms of entry, and other features of a particular virus

The most common application of iPSC-derived cells is disease modeling.^2,259,260^ A key advantage of the iPSC technology for modeling human diseases is that iPSCs can be derived from somatic cells of patients afflicted with a particular disease and carrying causal disease mutations or genetic risk factors. Such iPSCs with a disease-relevant genetic background are subsequently differentiated into the affected cell types that can reveal disease-specific phenotypes. For example, neurons differentiated from iPSCs of patients with familial Alzheimer’s disease recapitulate amyloid β pathology, tau phosphorylation, and other phenotypes observed in Alzheimer’s disease patients.^261–263^ Alternatively, disease-relevant mutations can be introduced by CRISPR/Cas9-based gene editing, which enables derivation of isogenic disease models.^264^ Isogenic cell lines can be generated by correcting disease-causing mutations in patient-derived iPSCs to obtain a wild-type control iPSC line.^265^ The resulting pair of patient-derived iPSCs and corrected control iPSCs shares the same genetic background except for the disease-causing mutation or genetic risk variant.^265,266^ For example, astrocytes derived from iPSCs of patients with Alexander’s disease reveal disease-specific phenotypes caused by *GFAP* mutations, whereas isogenic gene-corrected controls exhibit normal cellular function (Fig. 3a). Similarly, iPSC-derived astrocytes that carry the C variant of the rs11136000 SNP of the *CLU* gene, a known genetic risk factor for Alzheimer’s disease, but not isogenic SNP-corrected controls, negatively affect oligodendrocyte progenitor cell (OPC) proliferation and myelination.^267^ Using isogenic cell lines limits confounding individual-to-individual variation and may increase the statistical power of in vitro experiments.^268^ On the other hand, derivation of iPSCs from large cohorts of patients enables genome-wide association studies (GWAS) combined with phenotypic analysis.^269^ For example, analysis of iPSC-derived cortical neurons derived from a large cohort of Alzheimer’s disease patients reveals single-nucleotide polymorphisms (SNPs) associated with amyloid β production. Similarly, liver organoids derived from multiple donors reveal pleiotropic SNP interactions associated with non-alcoholic steatohepatitis (NASH).^269,270^ These iPSC cohorts can also be used to perform high-content screening to rapidly detect and compare disease-relevant pathology as well as evaluate therapeutic candidates.^271^ Establishing iPSC biobanks that contain multiple iPSC lines representing different diseases is thus an important goal for advancing iPSC-based disease modeling.

Given the multitude of disease modeling applications using iPSC-derived cells, the breadth of the relevant research could not be covered in a single review article. In the following sections, we consider several diseases that illustrate both the versatility of the iPSC platform as well as the different advantages and limitations of using iPSC-derived disease models. In particular, we discuss iPSC-based modeling of neurodevelopmental, psychiatric, and neurodegenerative diseases that are poorly recapitulated in animal models, require hard-to-access cell types, and can be age-related; cancer initiation that is difficult to study using primary cancer cell models that have already undergone transformation; and COVID-19 that illustrates rapid repurposing of iPSC-based cellular models to study a novel infectious disease during the height of a pandemic.

### Modeling neurodevelopmental and psychiatric disorders with iPSC-derived cells

Neurodevelopmental and psychiatric disorders are unique in that their pathogenesis manifests in cognitive changes that can only be studied using animal models that exhibit cognition, whereas in vitro experiments reveal molecular and cellular disease phenotypes only.^272,273^ However, neurological disorders, especially those that lack clear genetic etiology, cannot be easily recapitulated in animal models due to substantial species divergence and immense complexity of the human brain.^274–277^ These limitations have inevitably hindered scientific discovery and therapeutic development for neurological disorders. Nonetheless, iPSC-based cellular models can provide important insights into the pathogenesis of neurological disorders, whereas state-of-the-art technologies, such as brain organoid transplantation in vivo and machine learning, pave the way for studying complex cognitive phenotypes.

Neural cells derived from iPSCs of patients with neurological disorders exhibit impaired cellular function.^260,278^ For example, cellular models of schizophrenia reveal aberrant proliferation and migration of neural progenitor cells, dysfunctional arborization of cortical interneurons, and impaired astrocyte glutamate uptake.^279–283^ Neural progenitor cells derived from iPSCs of patients with the autism spectrum disorder (ASD) exhibit increased proliferation and impaired migration, as well as increased DNA damage and dysregulated chromatin accessibility at the molecular level.^284,285^ Various assays can be used to assess neuronal network connectivity in cell culture, which is used as a proxy for cognitive dysfunction. Synaptic density can be evaluated by immunostaining, whereas electrophysiology experiments, such as multi-electrode array (MEA)-based assays, can be applied to measure neuronal activity.^286–288^ Neuronal cultures derived from iPSCs of patients with schizophrenia exhibit decreased synaptic puncta density, defective glutamatergic synaptic transmission, and molecular phenotypes related to synaptic dysfunction.^289,290^ On the contrary, neuronal cultures derived from iPSCs of patients with ASD exhibit increased synaptic puncta density and neuronal firing rate, indicating neuronal hyperexcitability.^291^ Recently, MEA has also been combined with machine learning to create simulated environments, where neural cell cultures perform complex tasks and undergo synaptic remodeling—an in vitro assay for learning.^292,293^ It will be interesting to determine whether neurons derived from iPSCs of patients with neurological disorders exhibit impaired synaptic remodeling in such simulated environments.

Neurological disorders can also be modeled with brain organoids that can reveal dysfunctional cell-cell interactions and complex disease phenotypes.^294–297^ For example, brain organoids derived from iPSCs of patients with Down syndrome or ASD exhibit dysregulated proliferation of neural progenitor cells and aberrant production of inhibitory GABAergic interneurons.^298,299^ An important advantage of using brain organoids for the study of neurological disorders is their complex electrophysiological phenotypes that emerge as a result of improved neuronal maturation and 3D configuration.^300,301^ For example, cortical-ganglionic eminence assembloids derived from iPSCs of patients with Rett syndrome exhibit neuronal hyperexcitability and epileptiform-like activity characteristic of Rett syndrome.^302^ Finally, transplantation of iPSC-derived cells into the rodent brain allows the evaluation of cell behavior in a complex in vivo environment as well as cognitive dysfunction associated with the disease. For example, glial progenitor cells derived from iPSCs of patients with schizophrenia exhibit impaired astrocytic and oligodendrocytic differentiation, premature cell migration into the cortex, and hypomyelination.^303^ The chimeric mice also exhibit behavioral deficits, such as excessive anxiety, indicating higher-order neuronal network dysfunction.^303^ A powerful approach of iPSC-based modeling of neurological disorders is whole brain organoid transplantation in vivo, which not only creates a complex physiological milieu for the transplanted human cells, but also preserves human cell-specific organoid environment.^218,220,222,223,304,305^ Although neurological disorders have successfully been modeled using brain organoids in vitro, one important limitation of the brain organoid technology is their lack of vascularization, leading to poor nutrient and oxygen exchange, cellular stress, necrosis of the organoid core, and incomplete organoid maturation.^306^ Remarkably, brain organoid transplantation in vivo promotes robust organoid vascularization by the host vasculature and substantially improves organoid characteristics, including neuron maturation and microglia survival.^218,220,222,223,304,305^ An in vivo brain organoid model of Timothy syndrome reveals abnormal neuronal morphology and increased frequency of excitatory postsynaptic potentials, whereas a model of ASD indicates microglia activation.^220,222^ Overall, iPSC-derived cellular models of neurological disorders reveal complex molecular, cellular, and electrophysiological disease-related phenotypes.

### Modeling neurodegenerative diseases with iPSC-derived cells

**Fig. 4:**
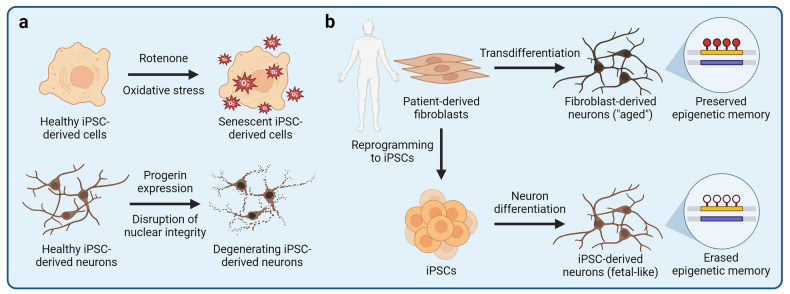
Modeling aging-associated phenotypes with iPSC-derived cells. One important limitation of using iPSC-derived cells to model human diseases is their fetal-like phenotypes and the lack of aging-associated cellular features. The process of somatic cell reprogramming to iPSCs is associated with a nearly complete erasure of aging-associated epigenetic marks and phenotypes. Therefore, various strategies to induce aging-associated phenotypes in iPSC-derived cells have been developed. **a** Exposure of iPSC-derived cells to compounds that disrupt cellular homeostasis can be used to induce aging-associated phenotypes, such as mitochondrial stress or cellular senescence. For example, rotenone disrupts electron transfer in mitochondria, leading to an increased production of reactive oxygen species that can cause mitochondrial stress, damage other organelles, and induce cellular senescence. **b** Aging-associated phenotypes can also be induced by ectopic expression of progerin, a truncated variant of lamin A nuclear lamina protein. Progerin causes the Hutchinson-Gilford progeria syndrome, a disease that manifests as accelerated aging due to the disruption of the nuclear lamina. Ectopic expression of progerin is sufficient to induce senescence- and aging-associated phenotypes in iPSC-derived neurons and other cells. **c** Aging-associated phenotypes are preserved if target cells are derived by direct transdifferentiation without an iPSC intermediate. Primary fibroblasts can be transdifferentiated into neurons that exhibit aging-associated phenotypes and epigenetic age signatures of the fibroblast donor, and can thus be used to study age-related dysfunction of neural cells

A distinct group of neurological disorders are age-related neurodegenerative diseases, such as Alzheimer’s disease, Parkinson’s disease, ALS, and others.^307–310^ In addition to various mutations and genetic risk factors, aging is a strong risk factor for such diseases and is tightly linked to their molecular mechanisms of progression.^311–313^ However, iPSC-derived cells are fetal-like and do not naturally exhibit aging-associated phenotypes.^314,315^ Somatic cell reprogramming to iPSCs is associated with cellular rejuvenation, causing the loss of aging-associated phenotypes, which are not restored upon iPSC differentiation.^316,317^ The lack of aging-associated phenotypes is a major limitation of iPSC-derived cells for disease modeling. Nonetheless, various iPSC-based models of neurodegenerative diseases have been developed, and methods to study age-related events or induce aging-associated phenotypes are emerging (Fig. 4).^306,314,318,319^

A small proportion of cases of age-related neurodegenerative diseases are familial in nature and are driven by genetic mutations. Such causal mutations are highly penetrant and manifest in clear molecular and cellular phenotypes of iPSC-derived cells. For example, cortical neurons carrying mutations in the *PSEN1* gene exhibit amyloid β pathology characteristic of Alzheimer’s disease^262^; dopaminergic neurons carrying mutations in the *SNCA* gene exhibit α-synuclein aggregation characteristic of Parkinson’s disease^320^; and motor neurons carrying mutations in the *TDP-43* gene exhibit TDP-43 aggregation characteristic of ALS.^321^ However, most cases of neurodegenerative diseases are sporadic and do not have a clear etiology. Various genetic risk factors for sporadic neurodegenerative diseases have been identified through GWAS, and their subtle contributions to disease progression can be modeled with iPSC-derived cells.^266,322–324^ For example, the E4 variant of the *APOE* gene is the strongest genetic risk factor for Alzheimer’s disease.^324–326^ Accordingly, iPSC-derived *APOE4* neurons, astrocytes, oligodendrocytes, and microglia, all exhibit dysregulated cellular homeostasis and function.^266,327–331^ Non-genetic effectors originating from outside the brain also influence progression of neurodegenerative diseases. Such effectors include the peripheral immune system that has recently been implicated in neurodegeneration as well as environmental factors, such as neurotoxins.^332–336^ For example, co-culture of iPSC-derived dopaminergic neurons with isogenic primary T cells isolated from patients with Parkinson’s disease reveals increased neuronal cell death that is mediated by T cell-secreted IL-17.^336^ Furthermore, exposure of iPSC-derived dopaminergic neurons to a neurotoxin 1-methyl-4-phenylpyridinium (MPP^+^) leads to increased expression of genes associated with Parkinson’s disease,^334^ whereas dopaminergic neurons carrying the A53T mutation in the *SNCA* gene are more susceptible to environmental pesticides than are normal controls.^335^ Finally, population level studies using large cohorts of iPSCs derived from patients with sporadic neurodegeneration may facilitate identification of novel biomarkers for patient stratification and reveal subtle genotype-phenotype relationships. Efforts to create disease-specific iPSC biobanks are underway; for example, hundreds of iPSC lines from patients with ALS have been established as part of the Answer ALS project.^337,338^ Interestingly, motor neurons derived from iPSCs of patients with sporadic ALS cluster into distinct groups based on their heterogenous phenotypes, illustrating the application of iPSC-derived cellular models to improve patient stratification.^339^

The models described above, however, do not incorporate aging-associated disease phenotypes that play a critical role in neurodegenerative diseases. Due to the lack of suitable models, it remains poorly defined how aging interacts with other risk factors to drive neurodegeneration. At the molecular level, aging may be associated with epigenetic erosion and DNA damage that derail homeostatic gene expression programs, resulting in suboptimal cellular phenotypes and cellular senescence.^340–345^ In iPSC-derived cells, aging-associated phenotypes, such as mitochondrial dysfunction, can be induced experimentally to mimic age-related cellular dysfunction (Fig. 4a). For example, iPSC-derived cells can be treated with rotenone that interferes with the mitochondrial electron transport chain, leading to increased production of reactive oxygen species (ROS), mitochondrial damage, and disruption of cellular homeostasis.^346–349^ However, it remains unclear whether disrupting one cellular pathway is sufficient to recapitulate aging or whether it is simply a model of cellular stress.^315^ An alternative strategy to induce aging-associated phenotypes is based on overexpression of progerin, a truncated variant of a nuclear lamina intermediate filament lamin A.^317^ Progerin is integral in the pathogenesis of Hutchinson-Gilford progeria syndrome (HGPS), a disease that causes premature aging.^350^ Remarkably, overexpression of progerin in iPSC-derived dopaminergic neurons induces neurite degeneration, neuromelanin accumulation, and aging-associated gene expression.^317^ Although progerin overexpression can induce various cellular phenotypes associated with aging, it should be noted that HGPS is a distinct disease that may not necessarily recapitulate normal human aging and may exhibit HGPS-specific phenotypes that are irrelevant to neurodegenerative diseases. Therefore, substantial efforts have been made to obtain human brain cell models without erasing aging-associated phenotypes of the somatic cells, from which the neural cells are derived. This aim can be achieved by direct transdifferentation of patient-derived fibroblasts into neurons without an iPSC intermediate (Fig. 4b).^351–356^ Fibroblasts can be transdifferentiated into neurons by overexpression of miRNAs or neuron fate-determining transcription factors, such as *NGN2* and *ASCL1*, combined with a small-molecule treatment.^354^ Transdifferentiated neurons retain the epigenetic age and aging-associated phenotypes of the fibroblast donor and can be used to study the impact of aging on the pathogenesis of neurodegenerative diseases.^316^ For example, transdifferentiated neurons derived from fibroblasts of elderly patients with Alzheimer’s disease reveal aberrant neuronal phenotypes, such as Warburg-like metabolic transformation, increased post-mitotic senescence, and hypo-mature neuronal identity, that are not observed in fetal-like iPSC-derived neurons.^316,357,358^ Finally, iPSC-derived cellular models can also be used to study age-related events by mimicking various cell non-autonomous conditions associated with aging. For example, breakdown of the BBB may be caused by aging and is a common feature of neurodegenerative diseases, leading to leakage of potentially neurotoxic serum components into the neural tissue.^359–363^ Mimicking the BBB breakdown by exposure of iPSC-derived brain organoids to human serum induces a rapid onset of Alzheimer’s disease-like pathology, including accumulation of amyloid β and phosphorylated tau as well as impaired neuronal activity (Fig. 3b).^364^ We anticipate that novel approaches to induce aging-associated phenotypes and model age-related events using iPSC-derived cells will provide new insights into both neurodegenerative and other age-related diseases.

### Modeling cancer initiation with iPSC-derived cells

Given their proliferative capacity, primary cancer cells derived from tumor biopsies are the most common cellular models for studying tumor cell biology and the response to therapeutic intervention.^365–367^ However, primary cancer cells have already undergone transformation, a key event that governs deregulation of cellular homeostasis and leads to cancer initiation.^368^ The iPSC technology offers a unique opportunity to study how various somatic mutations and other events rewire molecular and cellular programs of normal cells, so that they are transformed into cancer cells.^369^ For example, iPSC-derived neural stem cells carrying an H3.3K27M mutant histone H3.3 variant associated with diffuse intrinsic pontine glioma, a type of a juvenile brain tumor, exhibit aberrant gene expression programs that promote neural stem cell proliferation and stemness.^370^ Similarly, colonic organoids derived from iPSCs of patients carrying mutations in the *APC* gene associated with familial colorectal cancer exhibit elevated activity of the WNT signaling pathway and higher epithelial cell proliferation as compared to wild-type controls.^371^ In addition to somatic mutations, environmental factors also play a role in cell transformation. For example, chronic *Helicobacter pylori* infection is associated with increased incidence of gastric cancer, presumably due to persistent inflammation of the epithelial lining of the stomach.^372,373^ Injection of *H. pylori* bacteria into the lumen of iPSC-derived gastric organoids induces a rapid response of epithelial cells, including a twofold increase in cell proliferation.^374^ Finally, genetic manipulation of iPSCs and their subsequent differentiation into cancer-relevant cell types can be used to establish cancer evolution models that reflect successive acquisition of somatic mutations and clonal expansion of cancer cells. For example, introducing various driver mutations associated with acute myeloid leukemia into iPSCs followed by differentiation of hematopoietic progenitor cells enables modeling of leukemic transformation from premalignant cell states to transplantable leukemia.^375^ High-throughput profiling of gene expression across the continuum of leukemogenesis reveals distinct molecular pathways, such as dysregulated inflammatory signaling, that promote tumorigenesis.^375^ Overall, iPSC-based cellular models can provide important insights into molecular and cellular events governing cancer initiation, which may facilitate patient stratification for early screening and cancer prevention.

### Modeling COVID-19 with iPSC-derived cells

Modeling viral infection with iPSC-derived cellular models can reveal unique interactions between viruses and human cells (Fig. 3c).^288,376–379^ The COVID-19 pandemic caused by the severe acute respiratory syndrome coronavirus 2 (SARS-CoV-2) has prompted the scientific community to rapidly repurpose experimental platforms, so that SARS-CoV-2 cellular tropism, molecular mechanisms of entry, life cycle, and SARS-CoV-2 targeting therapeutics could be investigated.^380–382^ Although animal cell lines and models permissive to SARS-CoV-2 have been identified and developed, human iPSC-derived cellular models have the advantage of revealing human-specific SARS-CoV-2 tropism and vulnerabilities.^383–387^ Therefore, iPSC-based cellular models of SARS-CoV-2 infection have been swiftly applied to study COVID-19, revealing disease-specific phenotypes.^388^ For example, SARS-CoV-2 infection of iPSC-derived alveolar epithelial type 2 (AT2) cells cultured at air-liquid interface, a model for respiratory tract infection, induces cytotoxicity and a pro-inflammatory phenotype of AT2 cells.^389^ Co-culture of iPSC-derived macrophages and lung epithelial cells reveals a protective role of macrophages against the SARS-CoV-2 infection of epithelial cells; however, M1 and M2 polarized macrophages exhibit different inflammatory responses.^390^ Given widespread extrapulmonary manifestations of COVID-19,^391–393^ permissiveness of different tissues and organs to SARS-CoV-2 can be evaluated using tissue-specific organoids.^388,394^ For example, SARS-CoV-2 infects and productively replicates in salivary gland organoids, indicating the potential role for salivary glands as a reservoir of SARS-CoV-2.^221^ Similarly, SARS-CoV-2 actively replicates in capillary organoids, which may explain SARS-CoV-2-associated viremia.^395,396^ SARS-CoV-2 infection also induces cytotoxicity in iPSC-derived cardiomyocytes and cardiospheres, causing myofibrillar disruption, impaired cardiomyocyte beating, and cell death.^397–399^ Neurological manifestations of COVID-19 have also been documented.^400–402^ SARS-CoV-2 infects iPSC-derived neural progenitor cells, neurons, astrocytes, and brain organoids.^403–406^ SARS-CoV-2 infection of neural tissues leads to increased tau hyperphosphorylation, a hallmark of Alzheimer’s disease, suggesting that SARS-CoV-2 infection may have long-term neurological effects that could contribute to the onset of neurodegeneration.^403,407^ Interestingly, the susceptibility of iPSC-derived neurons and astrocytes to SARS-CoV-2 infection is dependent on the *APOE* variant; *APOE4* cells exhibit increased susceptibility to SARS-CoV-2 infection as compared to *APOE3* cells.^406^ Overall, the iPSC technology has been rapidly adapted to investigate human-specific disease phenotypes of COVID-19, providing vital insights into this life-threatening disease.

## Drug development using iPSC-derived cells

Various advantages of iPSC-derived cellular models discussed throughout this review are also applicable to drug development applications.^408^ Given their human origin, iPSC-derived cells can be used as a preclinical platform to test drug efficacy and toxicity as well as uncover human-specific molecular mechanisms of drug action. Various somatic cell types, including those that are inaccessible from primary sources, can be derived from disease-specific iPSCs that harbor relevant causal mutations or genetic risk variants to assess drug efficacy in the context of a specific genetic background. iPSC-based experiments can also be scaled to perform high-throughput drug screening with thousands of small-molecule candidates. For example, Gu et al. performed a survival screen of 4500 compounds based on the caspase 3/7 activity to identify anti-apoptotic compounds that limited death of endothelial cells derived from iPSCs of patients with pulmonary arterial hypertension.^409^ When combined with high-content imaging technologies, drug screening assays can be used to evaluate complex phenotypes, such as changes to cellular morphology or accumulation of disease-associated protein aggregates.^271,410,411^ Park et al. developed a high-throughput drug screening pipeline to evaluate amyloid β and tau pathology in brain organoids derived from iPSCs of patients with Alzheimer’s disease.^271^ In particular, the authors used tissue-clearing techniques and high-content imaging to visualize and quantify the burden of amyloid β and phosphorylated tau upon drug treatment.^271^ Combining iPSC-based drug screening with computational analyses and machine learning can reveal targetable regulatory nodes associated with a specific disease as well as therapeutic candidates for drug repurposing.^412^ Taubes et al. performed an in silico drug repurposing analysis to identify candidates that could reverse *APOE4*-associated gene expression signatures in Alzheimer’s disease.^413^ Having identified bumetanide as a potential candidate, the authors validated its efficacy in iPSC-derived *APOE4* neurons.^413^ Furthermore, Theodoris et al. used machine learning to identify small-molecule compounds that could reverse aberrant gene expression associated with haploinsufficiency for the *NOTCH1* gene in calcific aortic disease.^414^ The authors screened over 1500 predicted candidates using iPSC-derived endothelial cells and identified an inverse agonist of the estrogen-related receptor α (ERRα) as a potent hit.^414^

iPSC-derived cellular models can also be used to evaluate drug toxicity, which is a major cause of drug attrition in therapeutic development.^415,416^ Although preclinical toxicology is based on animal studies, human-specific drug toxicity may not necessarily manifest in animal models, leading to costly drug withdrawals late in the drug development pipeline. Therefore, the iPSC technology can be used as a complementary platform to assess drug toxicity and its human-specific molecular mechanisms.^417–419^ For example, drug nephrotoxicity may be evaluated using iPSC-derived podocytes that form the epithelial lining of the kidney glomerulus.^420^ A microfluidics-based glomerulus-on-a-chip recapitulates adriamycin-induced podocyte injury and albuminuria.^421^ Similarly, iPSC-derived 3D cardiac tissues recapitulate doxorubicin-induced cardiotoxicity, leading to disruption of sarcomeres and cessation of beating.^422^ Evaluating drug toxicity using patient-specific iPSCs may also facilitate precision medicine-driven patient stratification based on individual patient susceptibility to particular therapeutics. For example, transcriptomic analysis of a panel of iPSC-derived cardiomyocytes reveals patient-specific cardiomyocyte susceptibility to oxidative stress associated with decreased expression of the *NFE2L2* gene.^423^ Cardiomyocytes with low *NFE2L2* expression are more susceptible to tacrolimus- and rosiglitazone-mediated cardiotoxicity as compared to cardiomyocytes with high *NFE2L2* expression.^423^ Uncovering the mechanisms of drug toxicity can facilitate the development of novel therapeutic strategies to mitigate such toxicity. Sharma et al. found that exposure of iPSC-derived cardiomyocytes to cardiotoxic tyrosine kinase inhibitors leads to compensatory insulin signaling that may be cardioprotective.^424^ Indeed, adding exogenous insulin or IGF1 improves cardiomyocyte viability in the presence of tyrosine kinase inhibitors.^424^ Finally, drug toxicity can be elicited by unexpected drug distribution or accumulation in certain human tissues. Drug pharmacokinetics can be assessed in barrier-forming organoids, such as choroid plexus organoids that form fluid-filled cysts and exhibit selective permeability to various drugs.^425^ Drug absorption and metabolism by the cytochrome P450 (CYP) family enzymes can be evaluated using iPSC-derived intestinal epithelial cells.^426^ Humanized animal models can also reveal human tissue-specific drug pharmacokinetics and accumulation; for example, transplantation of iPSC-derived kidney organoids into athymic rats has been used to evaluate organoid exposure to systemically administered drugs.^427^ Overall, the iPSC technology enables complementary evaluation of drug efficacy and toxicity using human-specific models.

## iPSC-based cell therapy

Cell therapy has recently emerged as a promising approach to repair or replace damaged tissue as well as engineer immune responses to a disease, such as cancer.^428–433^ The success of adoptive chimeric antigen receptor (CAR) T cell therapy to treat acute lymphoblastic leukemia and large B cell lymphoma has paved the way for developing novel cell therapies, including those based on the iPSC technology.^11,434–436^ Although primary cells, such as T cells, natural killer (NK) cells, and mesenchymal stem cells, can be isolated from a patient and later used as autologous cell therapy, other cell types, such as neurons, cannot be harvested for transplantation. Furthermore, the quality of primary cells may be compromised by a disease or by germline mutations as well as exhibit unwanted heterogeneity. The iPSC technology can be used to overcome these limitations, given that iPSCs can be genetically engineered, clonally expanded, and differentiated into most somatic cell types.^11^ Furthermore, iPSC-based cell therapy has fewer ethical constraints as compared to ESC-based cell therapy because iPSCs are derived from somatic cells.^437,438^ Xenotransplantation experiments serve as a proof of principle that transplanted iPSC-derived cells can mitigate disease-associated tissue dysfunction and restore homeostasis. For example, transplanted human iPSC-derived pancreatic islets secrete insulin and control glycemia in diabetic mice^439^ and macaques.^440^ Similarly, human iPSC-derived OPCs rescue myelination in myelin-deficient mice upon transplantation, indicating the potential application of OPC-based cell therapy for treating demyelinating white matter disorders.^123,145,441^ These examples indicate that the iPSC technology can be used to derive hard-to-access cell types and restore normal tissue physiology upon transplantation. As a result, various clinical trials using iPSC-derived cellular products to treat human diseases have been initiated (Table 1).

**Table 1:** Selected interventional clinical trials of iPSC-based cell therapy

Organ	Indication	Cell Product	Phase	Study Type	Primary Outcome	Location	Trial ID
Brain	Parkinson’s disease	Allogeneic iPSC-derived dopaminergic progenitor cells	Phase I/II	Single-arm, open-label	Safety & graft expansion	Japan	JPRN-jRCT2090220384
		Allogeneic iPSC-derived dopaminergic progenitor cells	Phase III	Single-arm, open-label	Non-rejection rate	Japan	JPRN-jRCT2091220385
		Autologous iPSC-derived dopaminergic progenitor cells	N/S	Crossover, non-randomized, single-blind	Safety	China	NCT06145711
	Chronic ischemic stroke	Allogeneic iPSC-derived neural progenitor cells	Phase I	Single-arm, open-label	Safety	China	NCT06299033
	Acute ischemic stroke	Allogeneic iPSC-derived endothelial progenitor cells	Phase I	Parallel, randomized, double-blind	Safety	China	NCT05993884
Spinal cord	Spinal cord injury	Allogeneic iPSC-derived neural progenitor cells	Phase I/II	Single-arm, open-label	Safety	Japan	JPRN-jRCTa031190228
Eyes	Age-related macular degeneration	Allogeneic iPSC-derived RPE cells	N/S	Single-arm, open-label	Safety	Japan	JPRN-UMIN000026003
		Autologous iPSC-derived RPE cell sheet	N/S	Parallel, non-randomized, open-label	Safety	Japan	JPRN-UMIN000011929
		Autologous iPSC-derived RPE	Phase I/II	Single-arm, open-label	Safety & visual acuity change	United States	NCT04339764
		Autologous iPSC-derived RPE	Phase I	Single-arm, open-label	Safety	China	NCT05445063
	Retinitis pigmentosa	Allogeneic iPSC-derived retinal sheets	Phase I	Single-arm, open-label	Safety & increase in retinal thickness	Japan	JPRN-jRCTa050200027
	RPE-related impairment	Allogeneic iPSC-derived RPE cells	Phase I/II	Single-arm, open-label	Reduction of the window defect area	Japan	JPRN-jRCTa050200122
Bone marrow	Thrombocytopenia	Autologous iPSC-derived platelets	Phase I	Single arm, open-label	Safety	Japan	JPRN-jRCTa050190117
	Acute myeloid leukemia	Allogeneic iPSC-derived NK cells	Phase I	Sequential, non-randomized, open-label	Safety	United States	NCT04714372
Heart	Congenital heart disease	Autologous iPSC-derived cardiac lineage cells	Phase I	Parallel, non-randomized, open-label	Short term safety & feasibility	United States	NCT05647213
	Ischemic cardiomyopathy	Allogeneic iPSC-derived cardiomyocyte sheet	Phase I	Single-arm, open-label	Safety & improvement in LVEF	Japan	NCT04696328
	Heart failure	Allogeneic iPSC-derived engineered human myocardium	Phase I/II	Single-arm, open-label	Target heart wall thickness & heart wall thickening fraction	Germany	NCT04396899
		Allogeneic iPSC-derived cardiomyocyte spheroids	Phase I/II	Sequential, non-randomized, open-label	Safety	Japan	NCT04945018
		Allogeneic iPSC-derived cardiomyocytes	Phase I/II	Parallel, randomized, double-blind	Safety	China	NCT03763136
		Allogeneic iPSC-derived cardiomyocytes	Phase I	Parallel, randomized, double-blind	Safety	China	NCT04982081
Lungs	Respiratory failure	Allogeneic iPSC-derived mesenchymal stem cells	Phase I	Parallel, randomized, open-label	Assessment of respiratory dysfunction	Australia	NCT04537351
	COVID-19 in patients with hypoxia	Allogeneic iPSC-derived NK cells	Phase I	Sequential, non-randomized, open-label	Safety	United States	NCT04363346
Ovaries	Ovarian cancer	Allogeneic iPSC-derived NK cells	Phase I	Sequential, non-randomized, open-label	Safety	United States	NCT04630769
Systemic	Systemic lupus erythematosus	Allogeneic iPSC-derived CAR NK cells	Phase I	Sequential, non-randomized, open-label	Safety & recommended phase II regimen	United States	NCT06255028
	B cell cancers	Allogeneic iPSC-derived CAR NK cells	Phase I	Sequential, non-randomized, open-label	Safety & recommended phase II regimen	United States	NCT05336409
	Advanced solid tumors	Allogeneic iPSC-derived CAR T cells	Phase I	Parallel, non-randomized, open-label	Safety	United States	NCT06241456
	Advanced solid tumors	Allogeneic iPSC-derived NK cells	Phase I	Parallel, non-randomized, open-label	Safety	United States	NCT03841110
	Steroid-resistant acute GvHD	Allogeneic iPSC-derived mesenchymal stem cells	Phase I	Sequential, non-randomized, open-label	Safety	Australia, United Kingdom	NCT02923375
	High-risk acute GvHD	Allogeneic iPSC-derived mesenchymal stem cells	Phase II	Parallel, randomized, quadruple-blind	Overall response rate	United States, Australia	NCT05643638

*CAR* chimeric antigen receptor, *COVID-19* coronavirus disease 2019, *GvHD* graft-versus-host disease, *HIV* human immunodeficiency virus, *iPSCs* induced pluripotent stem cells, *LVEF* left ventricular ejection fraction, *N/S* not specified, *NK* natural killer, *RPE* retinal pigment epithelium

### Autologous iPSC-based cell therapy

**Fig. 5:**
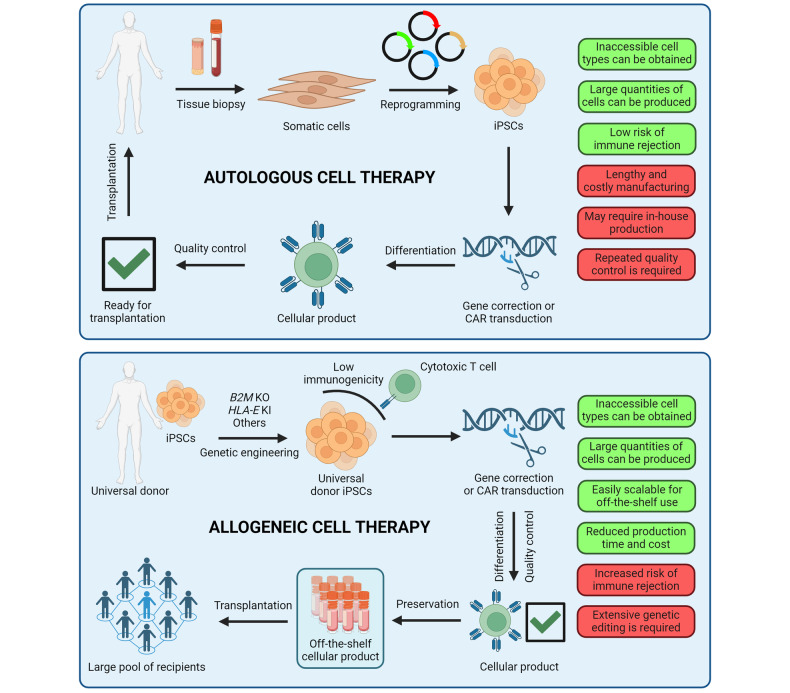
Autologous and allogeneic iPSC-based cell therapy. In autologous cell therapy, somatic cells are collected from the patient who will receive the cell transplant. The isolated somatic cells are reprogrammed into iPSCs, which can then be genetically engineered to correct disease-associated mutations or introduce new gene expression vectors. Modified iPSCs are differentiated into the cellular product that will be transplanted into the patient and rigorously evaluated for quality. In allogeneic cell therapy, iPSCs are taken from a biobank and genetically engineered for immune cloaking. The resulting hypoimmunogenic universal donor iPSCs can be further genetically modified to introduce cell therapy-specific gene expression vectors, such as a chimeric antigen receptor (CAR) expression cassette, and then differentiated into the desired cell type. After rigorous quality assessment, cellular products can be stocked and distributed as off-the-shelf therapeutics for transplantation into multiple recipients. KO, knockout; KI, knockin

**Fig. 6:**
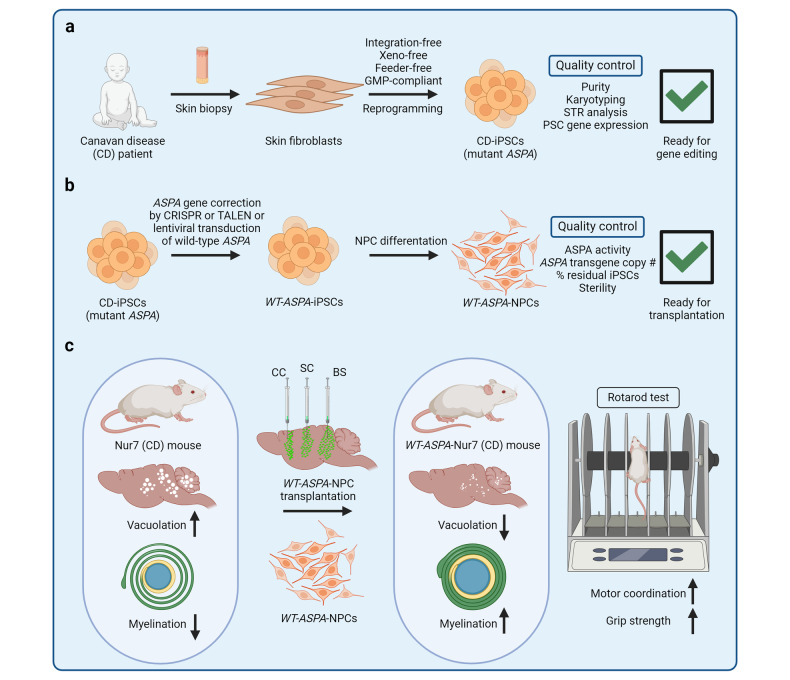
Development of iPSC-based autologous cell therapy. Despite the success of adoptive immune cell therapy, multiple other diseases affect cell types that cannot be easily isolated from patients for genetic engineering and transplantation back into the patient. For example, Canavan disease (CD) is a monogenic autosomal recessive neurological disorder caused by mutations in the aspartoacylase (*ASPA)* gene. These mutations disrupt ASPA enzymatic activity, leading to the accumulation of N-acetylaspartate (NAA) in the brain and causing spongy degeneration. ASPA enzymatic activity can be restored by transplantation of autologous neural progenitor cells (NPCs) that harbor CRISPR/Cas9-corrected *ASPA* or ectopically express wild-type *ASPA* delivered by lentiviral (LV) transduction. **a** A skin biopsy is obtained from a CD patient, and patient-specific iPSCs are derived from the isolated skin fibroblasts. **b** iPSCs are genetically engineered to restore wild-type *ASPA* expression and differentiated into NPCs that will be used for transplantation. **c** To demonstrate the efficacy of iPSC-derived NPC therapy for CD, preclinical experiments using a CD mouse model (Nur7) can be performed. CD mice exhibit characteristic spongy degeneration with vacuolation, myelin defects, and motor dysfunction. In our studies,^122,123,441^ we transplanted *WT-ASPA*-NPCs into the corpus callosum (CC), the subcortical region (SC), and the brainstem (BS) by stereotactic injection. We found that *WT-ASPA*-NPC-transplanted CD mice exhibited increased ASPA activity and reduced NAA levels, increased myelination and reduced vacuolation, and improved motor function. GMP, good manufacturing practice

iPSC-based cell therapy can be divided into two categories—autologous and allogeneic (Fig. 5). In autologous cell therapy, iPSCs are derived from the same patient who will receive the cell transplant.^442–444^ Autologous cell therapy is meant to prevent immune rejection of the transplant by the recipient because the immune system recognizes the transplanted cells as “self” tissue. A tissue biopsy is first collected from the patient who will undergo autologous cell therapy, and the isolated somatic cells are reprogrammed into iPSCs. These iPSCs can then be genetically modified to correct undesired mutations or introduce new gene expression cassettes. For example, if a patient has a monogenic disease that is caused by a germline mutation, gene correction can be performed. After genetic modification, iPSCs are differentiated into the desired cellular product that will be used for transplantation. Extensive quality control of iPSCs and iPSC-derived cells is required to ensure that the cellular product is functional and does not contain any deleterious or tumorigenic mutations. The feasibility of gene correction-based autologous cell therapy has been demonstrated in preclinical animal models. For example, transplantation of hepatocytes derived from gene-corrected iPSCs of a patient with hereditary antithrombin deficiency leads to normalization of antithrombin levels in the plasma of antithrombin-lacking mice, thus mitigating the thrombophilic state.^445^ Similarly, transplantation of pancreatic beta cells derived from gene-corrected iPSCs of a patient with monogenic Wolfram syndrome restores normal glucose homeostasis in diabetic mice.^446^ A detailed example of preclinical development of iPSC-based autologous cell therapy for Canavan disease, a monogenic neurodevelopmental disorder, is shown in Fig. 6.

### Allogeneic iPSC-based cell therapy

**Fig. 7:**
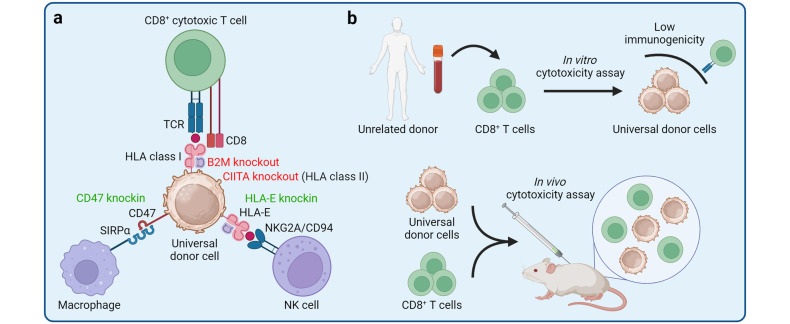
Engineering universal donor cells for allogeneic cell therapy. **a** Universal donor cells are genetically engineered to prevent the host immune response despite their foreign origin. CD8^+^ cytotoxic T cells recognize foreign cells via their T cell receptor (TCR) that interacts with human leukocyte antigen (HLA) class I molecules presenting unique antigens. If a foreign antigen is presented by the HLA class I molecules, CD8^+^ cytotoxic T cells initiate destruction of the encountered cell. Knockout of the β_2_ microglobulin (*B2M*) gene is sufficient to disrupt the universal donor cell interaction with CD8^+^ cytotoxic T cells. However, ablation of HLA class I molecules elicits a “missing-self” response by natural killer (NK) cells, leading to cell lysis. Therefore, *B2M* knockout is often combined with ectopic expression of HLA-E, which interacts with the inhibitory NK cell receptor NKG2A/CD94 to suppress the missing-self response. Knockout of *CIITA* disrupts foreign antigen presentation to CD4^+^ T cells via HLA class II molecules. To prevent macrophage-mediated cell killing, CD47 surface protein can be ectopically expressed in universal donor cells. CD47 interacts with the signal-regulatory protein α (SIRPα) and acts as the “don’t-eat-me” signal to suppress macrophage-mediated phagocytosis. **b** Hypoimmunogenicity of universal donor cells can be tested by performing in vitro and in vivo cytotoxicity assays, in which universal donor cells are mixed with primary immune cells, such as T cells, derived from an unrelated donor. Universal donor cells exhibit increased survival and stable persistence in the presence of primary immune cells of a mismatched donor, indicating successful immune evasion

In allogeneic cell therapy, iPSCs derived from a universal donor are used for transplantation, circumventing the lengthy and costly process of iPSC production from each patient who will receive the cell transplant (Fig. 5).^447,448^ The desired cells can be differentiated, characterized, and stocked in advance, so that the cellular product is available on demand or “off-the-shelf” without the need for in-house manufacturing. However, allogeneic cell therapy poses a risk of immune rejection and graft-versus-host disease, requiring additional “immune cloaking” strategies to evade the host immune system (Fig. 7a).^449^ Commonly used genetic modifications include knockout of the *B2M* gene, which encodes a component of human leukocyte antigen (HLA, also known as major histocompatibility complex, MHC) class I molecules, to disrupt foreign antigen presentation to cytotoxic CD8^+^ T cells; knockout of the *CIITA* gene to disrupt foreign antigen presentation to CD4^+^ helper T cells; overexpression of the B2M-HLA-E fusion construct to inhibit the “missing-self” response of NK cells; and overexpression of *CD47* to provide the “don’t-eat-me” signal to macrophages.^449^ A combination of such modifications is often used to evade different immune cell types. For example, Wang et al. engineered hypoimmunogenic universal donor iPSCs by knocking out *B2M*, *CIITA*, and *PVR* (encoding a ligand for NK cell activation) as well as overexpressing *B2M-HLA-E*.^450^ Hu et al. also knocked out *B2M* and *CIITA* but instead overexpressed *CD47*, having observed that not only macrophages but also most IL-2 stimulated NK cells present the SIRPα receptor of CD47.^451^ It should be noted that extensive genetic engineering required for immune cloaking can introduce off-target mutations, whereas prolonged iPSC culture and clonal expansion can lead to accumulation of spontaneous genetic aberrations. In our recent study, we knocked out *B2M* and *CIITA* and took advantage of endogenously expressed *CD47* in OPCs, our cell type of interest, to evade the NK response.^441^ Therefore, our approach requires two steps of genetic engineering only, reducing the likelihood of undesired mutational events. Having engineered the universal donor cells, their immune evasive properties can be validated in preclinical models. Universal donor cells and primary immune cells from an unrelated donor can be co-cultured together in vitro or co-injected in vivo to evaluate their survival and persistence (Fig. 7b).

An alternative approach to prevent immune rejection of allogeneic cell therapy is to establish HLA-homozygous iPSC haplobanks to match the donor-patient genotypes of the main HLA molecules involved in immune rejection.^452–455^ Several dozens of iPSC lines are sufficient to cover a large proportion of the population by HLA matching. For example, Yoshida et al. established a clinical-grade HLA haplobank of 27 iPSC lines derived from 7 donors, theoretically covering 40% of the Japanese population for HLA-matched iPSCs.^455^ Overall, allogeneic cell therapy holds great promise to streamline the production pipeline, but the safety concerns, especially those related to immune rejection, remain to be fully addressed.

### Challenges associated with iPSC-based cell therapy

Compared to pharmacological therapy, cell therapy is extremely complex and poses major safety, quality assurance, and logistical challenges, including those specific to iPSC-based therapeutics.^456,457^ A major concern is the propensity of iPSCs for teratoma formation; it is critical to ensure that undifferentiated iPSCs and stem cell-like intermediates are completely removed from the cellular product that will be transplanted into the patient to prevent tumor formation.^458^ Residual iPSCs can be removed from differentiated cell cultures by selective elimination of highly proliferative cells using chemotherapeutic drugs, such as doxorubicin,^459^ or by selective elimination of alkaline phosphatase-positive cells using toxic substrates of alkaline phosphatase.^460^ Introducing a gene encoding a self-destruction switch can provide an additional safety mechanism to selectively remove transplanted cells if they acquire tumorigenic properties.^461^ Such self-destruction systems include inducible activation of apoptosis, expression of enzymes that can convert non-toxic substrates into toxic compounds, and expression of surface receptors that can be targeted by infusion of monoclonal antibodies.^461^ As discussed earlier, iPSCs can also exhibit higher intrinsic genetic heterogeneity as compared to ESCs, and acquire mutations during reprogramming, prolonged culture, and gene editing.^111,462,463^ Such mutations may confer tumorigenic potential or lead to the emergence of novel immunogenic epitopes. Therefore, genetic analysis may be required at different stages of iPSC preparation to ensure that the cellular product is free of deleterious mutations.

Incomplete maturation of iPSC-derived cells remains a major hurdle in developing efficacious cell therapies. For example, iPSC-derived CAR T cells are often not as functional as CAR T cells derived from primary T cells, which may limit their tumor cell killing ability and persistence.^431,464^ Various approaches to improve iPSC differentiation and maturation protocols for cell therapy applications are under active investigation. For example, T cells can be differentiated using hematopoietic or thymic organoids that mimic the in vivo environment of the developing T cells.^465–467^ Challenges associated with efficacy of iPSC-based cell therapy for solid tissues include poor transplant engraftment and limited therapeutic response. Systemic infusion of cellular therapeutics may not be sufficient to establish a solid organ graft or may result in off-target engraftment.^456^ For example, intrasplenic infusion of iPSC-derived hepatocytes leads to their engraftment into various organs, including the liver, stomach, spleen, and large intestine.^468^ Engraftment can be controlled by using biomimetic scaffolds to differentiate cells as structured assemblies, followed by their direct transplantation into the recipient organ. Transplantation of iPSC-derived hepatocytes as a cell sheet generated using a supportive membrane promotes successful liver engraftment with no cells detected in other organs.^468^ Biodegradable scaffolds also promote integration and improve functionality of iPSC-derived retinal pigment epithelium patches as compared to epithelial cells cultured and transplanted without a scaffold.^469^ Similarly, bio-ink polymers with favorable rheological properties support osteogenic differentiation of iPSC-derived mesenchymal stromal cells and promote repair of cranial defects upon transplantation into a mouse model of cranial injury.^470^ Combination therapy can also improve the efficacy of iPSC-based cell therapy via synergistic mechanisms. For example, a combination therapy of iPSC-derived NK cells and anti-PD-1 immunotherapy synergize to kill tumor cells.^471^ Similarly, a combination therapy of the neurotrophic factor GDNF and iPSC-derived dopaminergic neurons to treat Parkinson’s disease results in brain-wide dopaminergic neuron innervation in a rat model, whereas transplantation of dopaminergic neurons alone is associated with poor long-distance innervation.^472^

Logistics, reproducibility, and the overall cost of iPSC-based cell therapies should also be considered. Logistical challenges include manufacturing and quality assurance of iPSC-based cell therapies.^457^ Off-the-shelf iPSC-derived cellular products for allogeneic cell therapy can be generated and distributed in a centralized manner, whereas autologous cell therapies might require hospital-affiliated personnel and facilities to routinely generate cellular products compliant with good manufacturing practices (GMP).^473^ Reproducibility and consistency of iPSC-derived cellular products can be improved by automating cell culture with liquid-handling robots, whereas large-scale differentiation of iPSCs can be achieved by using bioreactors. Stirred-tank bioreactors enable the scaling of suspension culture as well as monitoring of cell growth and various biophysical parameters, such as pH.^474,475^ Automation as well as optimization of iPSC derivation, maintenance, and differentiation protocols can also reduce the overall costs of iPSC-based cell therapies. For example, developing growth factor-free media formulations that do not require costly recombinant proteins could make iPSC maintenance more cost-effective.^476^ Although various challenges remain to be overcome, iPSC-based cell therapy holds great promise to restore tissue homeostasis and function in a way that cannot be achieved with pharmacological therapy.

## Concluding remarks and future perspectives

Since its development less than two decades ago, the iPSC platform has opened new frontiers for scientific discovery and therapeutic development. The study of somatic cell reprogramming has revealed immense complexity of cellular transformation that occurs during the induction of the pluripotent stem cell state and encompasses both deterministic and stochastic elements.^6^ These mechanisms have shed light on the central role of transcription factors in orchestrating gene expression programs, the importance of epigenetic regulation of cell fate, and the cooperative nature of different effectors of reprogramming. With increasing understanding of reprogramming mechanisms, novel methods for efficient and cost-effective derivation of iPSCs continue to emerge. For example, recent reports of fully chemical iPSC derivation methods hold promise for the development of fully defined, scalable, and rapid somatic cell reprogramming protocols.^128–130^

As in vitro models of human development, iPSCs and iPSC-derived cells have been used to investigate the principles and mechanisms of cell fate transitions, self-organization, and developmental disorders. Furthermore, iPSC-based cellular models for numerous other diseases, ranging from genetic to sporadic and age-related disorders, enable the study of human-specific disease mechanisms and the testing of potential therapeutic candidates in vitro.^2^ Sophisticated cellular models, including organs-on-a-chip, organoids, assembloids, and others, can be used to study higher-order tissue architecture, compartmentalization, and long-range interactions in human development and diseases.^10,160,163,188,477^ These advanced models of human tissues can also be used to evaluate drug efficacy, toxicity, and pharmacokinetics, thus serving as an additional preclinical platform for drug screening.^408^ We anticipate that the complexity and functional maturation of iPSC-derived cells and tissues will continue to improve and will reveal yet unappreciated mechanisms and phenotypes of human biology. For example, emerging methods for brain organoid transplantation and vascularization pave the way for obtaining highly functional and mature human cell-based neural tissues that can integrate into the host circuitry and influence animal behavior.^218,220,222^ Such models enable the study of neuronal network connectivity and its dysfunction in human-specific neurodevelopmental disorders that are challenging to reproduce in preclinical models.

Finally, the promise of the iPSC-based cell therapy has substantially materialized in the past decade, with numerous preclinical studies and early-stage clinical trials being conducted across the spectrum of human diseases (Table 1).^11^ These efforts are focused on various cancers, for which autologous and allogeneic iPSC-based immune cell therapies are being developed, genetic developmental disorders that require cell transplantation to restore tissue homeostasis, and even sporadic age-related diseases to replace degenerating tissues. Of notable interest are allogeneic cell therapies that utilize universal donor cells engineered to evade immune rejection.^448^ Universal donor cells can be prepared, characterized, and stocked in advance, considerably simplifying the manufacturing pipeline and reducing the turnaround time. Although important challenges associated with iPSC-based cell therapy remain to be resolved, the technology holds great promise to alleviate human diseases.

The technological advances that evolve alongside the iPSC technology offer new opportunities to define molecular mechanisms of iPSC induction, optimize protocols of iPSC differentiation into somatic cells, develop sophisticated drug screening platforms, and create efficacious cell therapies. We anticipate that improving technologies, such as microscopy tools,^478,479^ multiomics,^480^ CRISPR/Cas9-based studies of gene and protein function,^481–484^ epigenetic engineering,^485–488^ machine learning algorithms,^489–492^ and others, will provide new insights into the molecular events that govern somatic cell reprogramming to pluripotency and iPSC differentiation into terminal somatic cell types. The study of human development and diseases using iPSC-based models will benefit from enhanced collaboration, including the development of deeply characterized benchmark iPSC lines^493^ as well as ethnically diverse iPSC biobanks.^494^ Automation of iPSC differentiation into somatic cells and organoids will increase reproducibility of in vitro studies required for rigorous high-throughput applications, including drug screening.^495^ Finally, improving iPSC differentiation and maturation protocols will enable derivation of efficacious cellular products for therapeutic development, whereas production of entire iPSC-derived organs may be possible by chimeric organogenesis.^496–498^ Overall, the iPSC technology will continue to propel fundamental research and therapeutic development to accelerate scientific discovery and relieve human diseases.
